# Leveraging family-specific signatures for AMP discovery and high-throughput annotation

**DOI:** 10.1038/srep24684

**Published:** 2016-04-19

**Authors:** Faiza Hanif Waghu, Ram Shankar Barai, Susan Idicula-Thomas

**Affiliations:** 1 Biomedical Informatics Centre of Indian Council of Medical Research, National Institute for Research in Reproductive Health, Mumbai-400012, India

## Abstract

Antimicrobial peptides (AMPs) are diverse, biologically active, essential components of the innate immune system. As compared to conventional antibiotics, AMPs exhibit broad spectrum antimicrobial activity, reduced toxicity and reduced microbial resistance. They are widely researched for their therapeutic potential, especially against multi-drug resistant pathogens. AMPs are known to have family-specific sequence composition, which can be mined for their discovery and rational design. Here, we present a detailed family-based study on AMP families. The study involved the use of sequence signatures represented by patterns and hidden Markov models (HMMs) present in experimentally studied AMPs to identify novel AMPs. Along with AMPs, peptides hitherto lacking antimicrobial annotation were also retrieved and wet-lab studies on randomly selected sequences proved their antimicrobial activity against *Escherichia coli*. CAMPSign, a webserver has been created for researchers to effortlessly exploit the use of AMP family signatures for identification of AMPs. The webserver is available online at www.campsign.bicnirrh.res.in. In this work, we demonstrate an optimised and experimentally validated protocol along with a freely available webserver that uses family-based sequence signatures for accelerated discovery of novel AMPs.

Microbial resistance to conventional antibiotic treatment regimens is a growing health concern. Efforts to combat the threat posed by multi-drug resistant pathogens has accelerated the discovery and study of AMPs. AMPs are ancient, widely distributed, evolutionarily conserved effector molecules of the innate immune system and are studied for their potential as next generation therapeutics^1^. It is very difficult for microbes to circumvent AMPs, although few cases of resistance have been reported^2^. AMPs mostly target the membrane, which are quite recalcitrant to modification/redesign. Furthermore, AMPs exhibit pleotropic mechanisms of action such as inhibition of protein, nucleic acid or bacterial cell wall synthesis which further reduces the likelihood of AMP resistance^3^,^4^,^5^. These characteristics make AMPs likely candidates for discovery and design of anti-infective agents.

Identification of the precise antimicrobial region within the protein is a daunting task. Currently, bioinformatics-aided identification/prediction of AMPs is mostly restricted to knowledge-based prediction algorithms. Research on AMPs has shown that sequence composition of a peptide is an important determinant of its antimicrobial activity; minor changes in the residue information may render an antimicrobial peptide inactive^6^,^7^,^8^. Sequence composition of AMPs is heavily dependent on their families, for example, defensins contain conserved cysteines^9^, histatins are rich in histidines^10^ etc. This family-dependant sequence conservation can be leveraged for AMP prediction, however, studies that have exploited family-based information for AMP prediction are limited^11^,^12^.

Here, we present a detailed family-based study on AMP families. The study aims to accelerate the discovery of novel AMPs by exploiting the conserved sequence signatures present in AMP families. These signatures represented by patterns and HMMs were used to identify new AMP family members. The applicability of the method has been captured in CAMPSign, a webserver for large-scale identification of novel AMPs belonging to a particular AMP family, using family-specific signatures for the benefit of researchers.

## Results and Discussion

To validate the use of conserved residue information for AMP discovery, three AMP families were selected such that families that have both high and low level of sequence diversity are included. For e.g. β-defensins are present in a wide range of organisms and thus have low level of global sequence conservation whereas cecropins that are found majorly in insects (limited taxon) have well conserved sequences. A third family was selected to determine whether families with few members could be used for such studies e.g. transferrin (lactotransferrin).

### Generation and *in-silico* validation of family-specific patterns and HMMs

Signatures for cecropin and transferrin (lactotransferrin) family were obtained from CAMP_R3_ database^13^. CAMP_R3_ holds information on sequences, structures and family-specific signatures of prokaryotic and eukaryotic AMPs. It contains family-specific sequence signatures represented by patterns and hidden Markov models for 45 AMP families. While CAMP_R3_ contains signatures for defensin family, it does not contain signatures for the specific β-defensin family. Patterns and HMMs were thus generated for the β-defensin family using datasets A and B (see Methods). Patterns having fitness value of 26 or above were considered for further analysis (Table 1).

Family-specific signatures for cecropin, β-defensin and lactotransferrin families were validated by their precision/positive predictive value (PPV) and recall/sensitivity scores. These scores were calculated based on the annotation of the proteins which were retrieved by using these signatures as search queries. Protein sequences present in the manually curated SwissProt database^14^ having InterPro^15^ family descriptions were used for this analysis. Most of the signatures had good precision scores. The best signatures for cecropin, β-defensin and lactotransferrin exhibited as high as 100% precision and 98.68%, 50.44% and 66.66% recall/sensitivity scores, respectively.

The low recall/sensitivity values for β-defensin signatures are due to poor global sequence conservation within the different members of the β-defensin family. β-defensin is a large family and can be further subdivided into subgroups based on their source. Family-specific signatures created from such subgroups having higher overall sequence conservation are expected to enhance the recall/sensitivity of detection.

### Using patterns and HMMs for identification of novel AMPs

**Figure 1:**
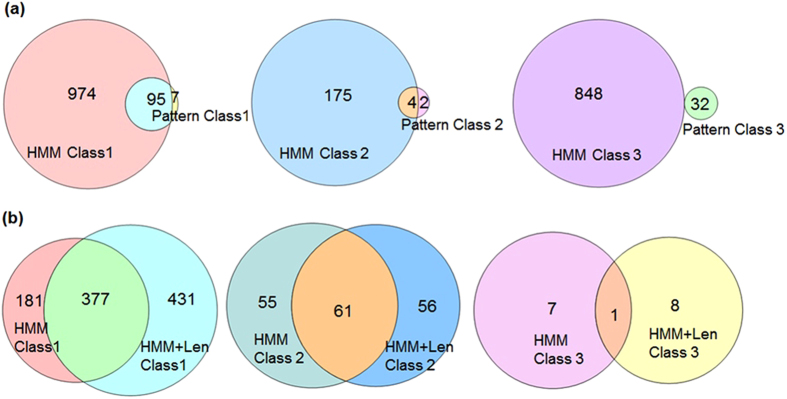
(**a**) Venn diagram of sequences retrieved using patterns and HMMs. **(b)** Venn diagram of sequences retrieved using length-based HMMs and HMMs created using sequences with undefined length.

As most of the family-based signatures exhibited good precision, these were queried against UniProt database^16^ to discover new AMP members. Proteins thus retrieved were categorized as “Class I”, “Class II” and “Class III” based on the definitions provided by UniProt. “Class – I” are AMPs; “Class – II” are uncharacterized proteins and predicted to be antimicrobial by either GO, InterPro or Pfam annotation and “Class – III” are proteins lacking antimicrobial definition or predicted activity. Accordingly, 1076 *Class I*, 181 *Class II* and 880 *Class III* proteins were retrieved (Fig. 1a). As expected, higher number of Class I proteins were retrieved. Class II and III proteins are promising candidates for AMP discovery. Albeit, presently lacking antimicrobial definition, experimental studies may confirm their antimicrobial activity (as can be seen in this study).

### Comparison between patterns and HMMs

HMMs retrieved more AMP sequences (Class I) as compared to patterns (Fig. 1a). However, patterns were much more specific as they retrieved fewer Class III sequences (lacking antimicrobial annotation) than HMMs (Fig. 1a). Thus, patterns and HMMs should be used in combination for optimal results.

### Importance of length-based signatures

Since sequence-based signatures are dependent on the length of the input sequences, dataset A was further subdivided into subgroups based on sequence length. HMMs created using these subsets were able to retrieve several more AMPs (431) that were missed by HMMs created using sequences with undefined length (Fig. 1b). Nevertheless, HMMs created using sequences with undefined length could identify few AMPs not retrieved by length-based HMMs. Thus, it is wise to incorporate length-based signatures along with signatures created using sequences of undefined length in the cocktail of search query to efficiently fish out all the members.

### Experimental validation of family-specific patterns and HMMs

Three sequences, retrieved using the selected AMP family signatures, were shortlisted for further experimental validation as proof of concept for the use of family-specific sequence signatures for AMP identification. One of these was retrieved using a pattern and belonged to the Class III category (lacking antimicrobial annotation) whereas the other two were retrieved using both types of HMMs (length-based HMM and HMM created using sequences of undefined length) and belonged to the Class II category (predicted as antimicrobial by either GO, InterPro or Pfam annotation). Antimicrobial regions, within these sequences, were identified based on either alignment with the corresponding patterns (lactotransferrin) or the predicted region based on homology (defensin and cecropin). These sequences were synthesized and tested for their antimicrobial activity (Table 2).

The MICs of the synthesized peptides against two strains of *E. coli* are shown in Table 3. LactP lacks activity against both the strains of *E. coli*, whereas the other two peptides, DefH and CecH are active against *E. coli*. CecH exhibits activity similar to that of gentamycin against both the strains of *E. coli*.

**Figure 2:**
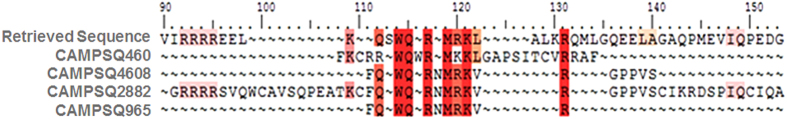
Pattern-restricted multiple sequence alignment of the sequence retrieved using pattern of lactotransferrin with the experimentally validated sequences of lactotransferrin.

LactP represents the part of the retrieved sequence corresponding to the pattern identified for lactotransferrin. Since pattern captures the most conserved region of AMPs within a family, it is highly likely that the precise AMP region may not be captured in the pattern. In order to refine/determine the antimicrobial region, the retrieved sequence was aligned with the experimentally validated AMPs of the lactotransferrin family (Fig. 2). Based on the alignment and sequence conservation, two sequences with additional flanking residues (LactP1, LactP2) were synthesized (Table 2). Antimicrobial activity of these two peptides was studied against both the strains of *E. coli* (Table 3). LactP1 exhibited 50% inhibition at 100 microM against both the strains of *E. coli* whereas LactP2 which had more number of flanking residues exhibited 100% inhibition at 100 microM against one strain of *E. coli*. It is highly likely that if more flanking residues are considered and the region is further refined, the antimicrobial activity of the resulting peptides may be significantly enhanced.

This experiment demonstrates that although patterns are very useful for identifying novel members of an AMP family, pattern sequence itself may/may not contribute to antimicrobial activity. The retrieved sequences should be aligned with experimentally validated sequences of the same family to identify/refine the antimicrobial region within the sequence.

### Creation and validation of a webserver for identification of novel AMPs based on AMP family signatures

A freely-available, user-friendly, online webserver named CAMPSign was developed to help researchers leverage the potential of family-based signatures for identification of novel AMPs. The tool utilizes family-specific signatures captured using patterns and HMMs for identification of peptides belonging to a particular AMP family. In contrast to the online prediction algorithms, such as CAMP^6^, APD3 (The antimicrobial peptide database)^17^ etc. which predict AMPs based on the physicochemical features such as charge, hydrophobicity, amphipathicity, helicity etc. which are known determinants for antimicrobial activity, CAMPSign predicts AMPs based on the presence of conserved AMP family-specific sequence composition. The input sequence/s are queried against family-specific signature database consisting of patterns and HMMs. Users can scan their sequence/s against all or a specific AMP family. Results, generated in a tabular format, indicate the family and number of patterns and HMMs that match to the user-defined sequence/s. Users can use the hyperlinks to visualise the detailed results for the patterns and HMMs that match/align to the sequence. Users can further query the selected sequences to search for identical or homologous sequences that are present in popular AMP databases^18^ using the basic local alignment search tool (BLAST) algorithm^19^.

Various measures of predictive performance such as recall/sensitivity, specificity, precision/positive predictive value (PPV) and negative predictive value (NPV) were calculated for validating the CAMPSign algorithm (Table 4). The algorithm exhibited higher specificity as compared to sensitivity.

### Highlights of the study

1. Family-specific signatures can be used for high-throughput identification of AMPs.
2. Patterns and HMMs should be used in combination for efficient retrieval of novel AMPs.
3. Length-based signatures help to retrieve AMPs missed out by signatures created using sequences with undefined length.
4. A protein with annotation in GenBank as ‘*Unknown secreted protein*’ (Accession: BAM18456) was experimentally demonstrated to belong to cecropin family with antimicrobial activity similar to gentamycin (antibiotic) when tested against Gram negative *E. coli*. The Class II and Class III peptides identified in this study using the family-based signatures are predicted to harbour antimicrobial activity. Researchers can assay the antimicrobial activity of these peptides for identification of novel AMPs. Due to limited funds, we have experimentally validated only 3 of the 1061 peptides available for experimental validation.
5. CAMPSign, the online webserver can aid in high-throughput identification of AMPs using family-specific signatures.

## Methods

### Data collection

Family-specific signatures for cecropin and lactotransferrin family were obtained from the CAMP_R3_ database^13^. Since, the database does not include family-specific signatures for β-defensins specifically; patterns and HMMs were generated for members of the β-defensin family. Representative AMPs which were experimentally validated and belonged to β-defensin family were retrieved from the CAMP_R3_ database to create dataset A.

These AMP sequences were further sub-divided based on their length. Groups were created for lengths ranging from 6–37 residues. These length-based groups constituted dataset B. Signatures were created for each subgroup of dataset B which contained two or more sequences.

### Sequence analysis

**Figure 3:**
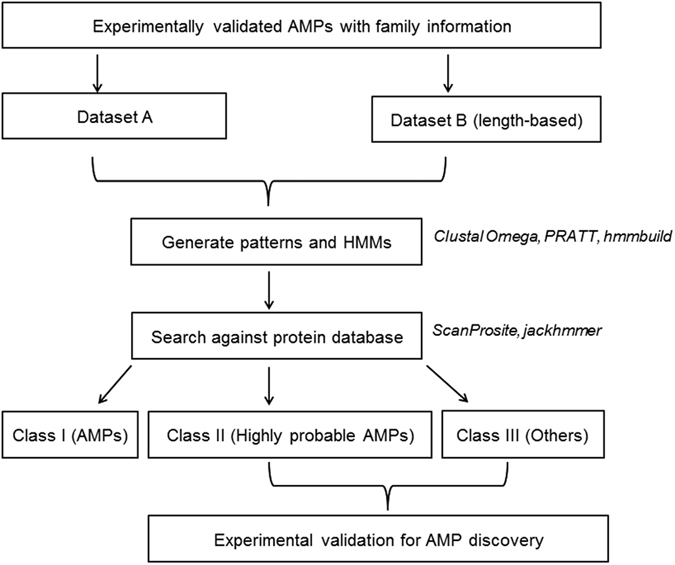
Flowchart of the protocol adopted for generation and use of signatures for AMP discovery.

- *Generation of patterns and pattern scan*: Patterns were generated for datasets A and B using PRATT tool^20^. Fitness value (score) of 26 or above was heuristically fixed as a threshold for selecting patterns for retrieval of sequences. These patterns were queried against the UniProt database^16^ using ScanProsite tool^21^ to retrieve related sequences.
- *Generation of HMMs and HMM search*: HMMs were generated for datasets A and B using ‘hmmbuild’ program of HMMER 3.1b1 package^22^. Multiple sequence alignments were created using Clustal Omega^23^,^24^. HMM models were built using the aligned sequences. These HMMs were searched against UniProt database^16^ to retrieve related sequences. For this, jackhmmer tool of HMMER web server^25^ was used with threshold e-value of 0.005 to increase the stringency. The search was run until convergence or stopped after three iterations.
- *In-silico validation of the patterns and HMMs*: Retrieved sequences that belonged to Swiss-Prot database^14^ and had InterPro family descriptions^15^ were considered for calculation of the precision/positive predictive value (PPV) and recall/sensitivity scores for the generated and obtained patterns and HMMs, as shown below.Precision/Positive predictive value (PPV) = True Positives/(True Positives + False Positives).Precision/Positive predictive value is the fraction of retrieved instances that are relevant to the query^26^. In this case, it is the fraction of AMPs (true positives) obtained in the search.Recall/Sensitivity = True Positives/(True Positives + False Negatives).Recall/sensitivity is the fraction of relevant instances that are successfully retrieved^26^. In this case, it is the probability of an AMP being correctly predicted as antimicrobial.For a given pattern/HMM, true positives are retrieved sequences that have the expected (as that of the pattern/HMM) InterPro family description; false positives are retrieved sequences that do not have the expected InterPro family description and false negatives are sequences with the expected InterPro family description in the SwissProt database that were not retrieved using the pattern/HMM.
- *Pattern-restricted sequence alignment*: To identify/refine the probable AMP region within the retrieved sequence, multiple sequence viewer of Schrödinger Suite Release 2014-2 (Schrödinger Release 2014-2: Prime, version 3.6, Schrödinger, LLC, New York, NY, 2014) was used to perform multiple sequence alignment of the experimentally validated AMPs belonging to a particular AMP family along with the retrieved sequence. This tool has an option to restrict the alignment based on a user-defined pattern. Pattern and the experimentally validated AMPs represent a particular AMP family, while the retrieved sequence is predicted to be a member of this family based on the pattern. Flowchart of the work implemented in this study can be viewed in Fig. 3.

### Peptide synthesis and antimicrobial assay

The selected sequences for experimental validation of the patterns/HMMs were chemically synthesized and purified by USV limited, Mumbai using fluoren-9-ylmethoxycarbonyl (Fmoc) solid-phase technique to study their antimicrobial activity. The peptides were terminally modified using N-terminal acetylation and C-terminal amidation.

Antimicrobial activity of the synthesized peptides was determined by broth microdilution assay and evaluated based on minimum inhibitory concentration (MIC). MIC is defined as the lowest concentration of peptide which completely inhibits the growth of test organisms. Gram negative microorganisms (*Escherichia coli* ATCC 8739, *Escherichia coli* ATCC 25922) were grown in Mueller-Hinton (MH) broth at 37 °C for 18h on a shaker to obtain an OD600nm of 0.8. Bacterial cultures were diluted in MH broth to obtain a cell density of 10^5^–10^6 ^c.f.u/ml. Different concentrations of peptides, ranging from 1–100 μM were serially diluted in MH broth and inoculated with bacterial cultures in 1:1 ratio in sterile 96-well microtiter plates. The plates were incubated at 37 °C for 18h and antimicrobial activity was evaluated as MIC of the peptides by determining the absorbance at 600nm. The microorganisms were obtained from National Collection of Industrial Microorganisms (NCIM), Pune.

### Creation and validation of CAMPSign tool

CAMPSign contains family-specific signatures obtained from CAMP_R3_^13^ for AMP family analysis. The webserver was developed using PHP 5, HTML, JavaScript, and Perl. An in-house script and command-line version of hmmscan (HMMER software)^22^ were used to scan sequences against pattern and HMM database respectively. BLAST algorithm^19^ was used to find homologous AMP sequences present in AMP databases^18^.

#### Validation of the CAMPSign tool

Creation of the test dataset: Positive dataset: Sequences that were a) annotated as “antimicrobial” by the Swiss-Prot database; b) belonged to one of the 45 AMP families present in the CAMP_R3_ database and c) absent in the training dataset used for generation of family signatures (patterns and HMMs) were included in the positive dataset. Of these, sequences which had ‘X’ in the residue information were excluded. The final positive test dataset contained 628 sequences.

Negative dataset: For creation of the negative dataset, sequences from the Swiss-Prot database with known function were selected. These were filtered to remove sequences present in the positive dataset and containing annotation as ‘antimicrobial’, ‘antibacterial’, ‘antifungal’, ‘antiviral’, ‘antiparasitic’, ‘defensin like’, ‘putative’ and ‘uncharacterized’. From this set of sequences, 2598 sequences were randomly selected using R statistical language^27^. The final negative test dataset comprised of 2579 sequences after removing sequences containing ‘X’.

Sequence filters were used to ensure that none of the training dataset sequences used for generation of patterns and HMMs were present in the test dataset.

**Figure 4:**
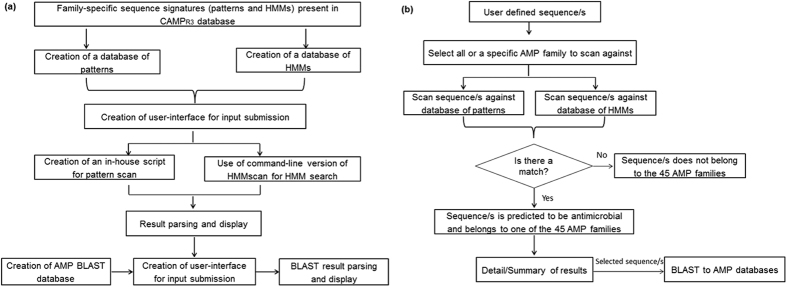
Flowchart of (**a**) design and (**b**) working of CAMPSign webserver.

The performance of the tool was measured in terms of recall/sensitivity, specificity, precision/ positive predictive value (PPV) and negative predictive value (NPV). Specificity is the probability of a non-AMP being correctly predicted as non-antimicrobial. NPV is the ratio of non-AMPs (true negatives) and all predicted non-AMPs. All values have been calculated as percentage. Flow chart of the design and working of CAMPSign can be seen in Fig. 4.

## Additional Information

**How to cite this article**: Waghu, F. H. *et al*. Leveraging family-specific signatures for AMP discovery and high-throughput annotation. *Sci. Rep*. **6**, 24684; doi: 10.1038/srep24684 (2016).

## Figures and Tables

**Table 1:** Patterns considered for members of cecropin, β-defensin and lactotransferrin families.

Family	Pattern	Sequence length used for pattern creation
β-defensin	C-x-C-x(0,2)-R-x(1,3)-C-x(2)-[GP]-x-R-R-x-G-T-C-[IL]-[FY]-Q-[GN]-R-[LV]-[WY]-[AT]-F-C-C	30
	C-x(0,1)-R-x(2,4)-C-[AIP]-x(2)-[EK]-x(3)-[GIL]-x(3)-[SV]-[GN]-x(4)-[FLV]-[HKR]	34
Cecropin	L-[ES]-K-[TV]-x-[KR]-x-[IL]-[ER]-N-[GS]-[AI]-x-[KR]-x(2)-[GS]-[EP]-[AG]-[IV]-A-[IV]-[AI]-[GI]-x-[AG]	31
	K-[ILV]-x-K-K-I-E-x(2)-G-x(0,1)-R-[NV]-[FIV]-[KR]-[ADN]-[AG]-x(3)-[AL]-[AGP]-[PV]-[AV]-[AIV]-[AEG]-V-x-[AG]-x-[AG]	35
	W-x-[LPV]-F-K-x(0,2)-E-x(2)-G-[QR]-[NR]-[IV]-R-[DN]-[AG]-[IV]-[IV]-x-A-[AG]-[AP]-[AV]-[AIV]-[AET]-[TV]-[GLV]-[GQ]-[AEQS]-A-x(2)-[AIL]	37
Lactotransferrin	W-Q-x(0,1)-R-x(0,1)-M-[KR]-K-[LV]	Undefined

**Table 2:** Peptides synthesised and assayed for antimicrobial activity.

Peptide	Family	Sequence	Length
DefH	Defensin	PLSCRRKIGICVLIRCSGNMRQIGTCLGALVKCCR	35
CecH	Cecropin	KIFKKIEKVGRNVRDGIIKAGPAVAVVEQA	30
LactP	Lactotransferrin	WQRMRKL	7
LactP1*	Lactotransferrin	KQS*WQRMRKL*ALKR	14
LactP2*	Lactotransferrin	RRRREELKQS*WQRMRKL*ALKR	21

*Lactotransferrin sequences refined based on multiple sequence alignment.

**Table 3:** Antimicrobial activity of the synthesized peptides.

Microorganisms	MIC (microM)^#^					
	DefH	CecH	LactP	LactP1	LactP2	Gentamycin
*E. coli* 25922 (NCIM 2931)	NA	6.25–12.5	NA	50–100*	50–100*	1.56–3.125
*E. coli* 8739 (NCIM 2065)	50–100*	3.125–6.25	NA	50–100*	50–100	1.56–3.125

^#^Minimal inhibitory concentration (MIC) was the average range of values obtained from triplicates of three independent experiments. NA: Did not show activity upto 100 microM. *50% inhibition.

**Table 4:** Predictive performance of the CAMPSign algorithm.

Measure of predictive performance	Formula*	Value/Score (%)
Recall or Sensitivity	TP/(TP + FN)	84.71
Specificity	TN/(TN + FP)	99.84
Precision or Positive predictive value (PPV)	TP/(TP + FP)	99.25
Negative predictive value (NPV)	TN/(TN + FN)	96.40

*TP is true positive, FP is false positive, TN is true negative and FN is false negative.
